# The Effects of Fixture Congestion on Injury in Professional Male Soccer: A Systematic Review

**DOI:** 10.1007/s40279-022-01799-5

**Published:** 2022-12-17

**Authors:** Richard Michael Page, Adam Field, Ben Langley, Liam David Harper, Ross Julian

**Affiliations:** 1 Department of Sport and Physical Activity, Edge Hill University, St. Helens Road, Ormskirk, Lancashire, L39 4QP UK; 2 Department of Sport and Exercise Sciences, Manchester Metropolitan University, Manchester, M15 6BH UK; 3 Department of Life Sciences, Manchester Metropolitan University, Manchester, M15 6BH UK; 4 Institute of Sport and Exercise Sciences, University of Muenster, 48149 Muenster, Germany; 5 School of Sport and Exercise, Exercise and Sport Research Centre, University of Gloucestershire, The Park, Cheltenham, GL50 2RH UK

## Abstract

**Background:**

Professional soccer teams are often required to compete with ≤ 4 days recovery between matches. Since congested schedules reduce recovery time between matches, players are possibly at an increased injury risk. To date, there are no published systematic reviews on the impact of match congestion on injuries during professional male soccer.

**Objective:**

The aim of this systematic review was to assess the effects of fixture congestion on injuries during professional soccer.

**Methods:**

Following pre-registration on the Open Science Framework (https://osf.io/86m25/) and conforming with Preferred Reporting Items for Systematic Reviews and Meta-Analyses (PRISMA) guidelines, systematic searches of four electronic databases (PubMed, Scopus, SPORTDiscus, and Web of Science) were conducted by independent researchers from inception until February 2022. Articles were included if they were original articles written in English and contained relevant time-loss injury data (injury that results in unavailability for training and/or match-play) for male professional soccer players regarding periods of fixture congestion (a minimum of two matches with ≤ 4 days recovery).

**Results:**

A total of eight articles were included in the review. Five studies identified that congested fixture schedules expose players to increased match injury incidence, although layoff duration was typically lower during congested periods. Two studies identified that training and overall injury incidence were higher during congested periods, with another study identifying a lower training injury incidence during congested periods.

**Conclusion:**

Injury risk is, overall, increased during fixture-congested periods; however, the layoff duration is typically shorter. The current findings have implications for practitioners regarding the management, periodisation, monitoring, and design of training and competition schedules.

**Supplementary Information:**

The online version contains supplementary material available at 10.1007/s40279-022-01799-5.

## Key Points

**Table:** 

Results suggest that overall (five from the eight included studies), congested fixture schedules expose players to increased match injury incidence, although layoff duration was typically lower during congested periods compared with non-congested periods.
The data in relation to training and overall injury incidence were somewhat equivocal, with two studies identifying that training and overall injury incidence were higher during congested periods, with another study identifying a higher training injury incidence during non-congested periods and no significant differences in overall injury incidence.
Except for injury incidence and layoff duration data, there is a lack of consistency in the reporting of injury data between studies. Fixture congestion is a contemporary and concerning issue (including to the players themselves) and more research is required to add further detail into the injury response observed during congested match schedules.

## Introduction

In modern professional soccer, clubs can compete in a large number of matches (50–80) during an approximately 40-week competitive season, which commonly involves participating in multiple matches (two to three) within a weekly microcycle [1, 2]. Individual players may be exposed to more than 10 consecutive weeks of a congested calendar (playing both domestically and internationally [3]), with some professional clubs exposed to 20+ weeks of fixture congestion across a competitive season. These congested match scenarios occur most frequently for elite players who compete in a number of domestic competitions in addition to continental and international tournaments [4]. Recent empirical evidence revealed that around 40% of professional soccer players perceive they are competing in an excessive number of matches per season [5], with 55% of players identifying that they have suffered at least one injury due to an overloaded schedule [3]. Under such conditions, the risk of injury could be exacerbated since insufficient recovery between successive matches and the occurrence of congested fixture periods have been previously identified as two of the top five extrinsic risk factors associated with soccer injuries in teams at the FIFA 2014 World Cup [6]. It has also been identified that a 2-day period is not sufficient to allow for full recovery between matches [7, 8]. Therefore, since congested fixture schedules reduce the number of recovery days between matches [9, 10], players repeatedly exposed to such intensified periods are possibly at an increased injury risk [11].

The risk of injury in professional soccer is well documented [6, 12–19], with multiple studies reporting on the incidence, layoff duration, and type and location of injuries [20–22]. Injuries in professional soccer can cost clubs in excess of £400,000 per injured player per month [23] and player availability is associated with overall team success (league position, matches won, goals scored, total points) [24, 25]. Professional soccer teams typically suffer around two injuries per player during a season [21], with injury occurrence during matches (36 injuries/1000 h of exposure) reportedly 10 times higher when compared with training (3.7 injuries/1000 h of exposure) [12]. The proposed aetiological risk factors for injury incidence in professional soccer players are limited flexibility [26, 27], muscle strength deficits or imbalances [27, 28], fatigue (muscle injury rates increased towards the latter stages of match-play) [29–31], increases in sprinting activity during matches [32], and increased competition/match loads [33, 34]. Therefore, considering the financial and success implications associated with injury and the frequency of injury incidence across a season, evaluation of injury risk across successive matches, especially during periods of fixture congestion, is important.

Previous research suggests that running profiles are similar between matches during congested schedules [35, 36], but injury propensity is increased during the second and third matches of a weekly microcycle [1, 36]. A recent systematic and meta-analytical review identified that although overall distances remain unimpacted, differences were observed across congested matches at moderate and lower intensities versus non-congested periods [2]. This suggests that players may subconsciously adopt pacing approaches to maintain high-speed running performance and avoid injury. In support of this, reductions in eccentric knee flexor strength and lower limb muscle activation are exacerbated by repeated bouts of standardised treadmill-based soccer-specific exercise with minimal recovery (simulating fixture congestion) [37]. These findings suggest that potential markers of injury risk are elevated during simulated periods of fixture congestion and may explain a possibly increased injury incidence within these periods. It is also plausible that recovery could be further impaired within a congested schedule due to travelling to and from away matches [38, 39] or playing at night [40]. Therefore, it is key that the extent to which fixture-congested schedules affect injury susceptibility during matches is fully understood.

In addition to understanding differences in injury incidence across congested and non-congested schedules, there is also merit in considering additional measures associated with the injuries suffered, such as, but not limited to, the mechanism, location, the timing, and time lost due to injury. These additional details will provide increased specificity to inform applied practice. Likewise, when considering the different approaches taken by clubs to manage their perceived increase in workload during congested schedules, the injuries suffered during these periods may differ in characteristics to those suffered during non-congested periods. One issue faced when comparing epidemiological research is the inconsistency in injury surveillance approaches and how this can influence injury data. As such, the ability to make specific comparisons between congested and non-congested periods may be influenced by the homogeneity of methods as well as the way in which certain metrics were reported and defined.

Although a recent systematic review and meta-analysis was conducted in this area [2], the article was focused on the impact of fixture congestion on performance. However, to date, there are no systematic reviews published that have attempted to review previous literature on injury incidences during congested match schedules. Therefore, systematic and critical appraisal of the literature documenting the effects of fixture congestion on injuries is required. The aim of this systematic review was to assess the effects of fixture congestion on injuries during professional male soccer. Suggestions will also be provided to improve future practices and inform future research opportunities.

## Methods

A systematic review was conducted to evaluate the influence of fixture congestion on injuries in professional male soccer. The current study was conducted and reported in accordance with the Preferred Reporting for Systematic Reviews and Meta-Analyses (PRISMA) statement (http://www.prisma-statement.org). The protocol was preregistered on the Open Science Framework prior to searches and analyses being completed (https://osf.io/8dsvw).

### Selection Criteria

To be included within the systematic review, studies were required to fulfil the following criteria: (1) original article was written in English; (2) abstracts were available for screening; (3) relevant data regarding periods of fixture congestion on injuries during soccer match-play; (4) injury that results in unavailability for training and/or match-play; (5) must contain data on a congested period defined as a minimum of two matches with ≤ 4 days recovery between successive matches; and (6) included professional male soccer players. There were no restrictions in terms of publication date. Manuscripts were omitted if they violated any of the following criteria: (1) inclusion of female or non-professional soccer players; (2) data only assessed the impact of congestion on performance and technical and tactical responses; (3) published in formats other than original research studies in peer-reviewed journals.

### Search Strategy

To identify suitable articles for the current systematic review, literature searches were conducted in PubMed, Scopus, SPORTDiscus and Web of Science. All searches were conducted in February 2022 by two of the authors (RMP and RJ). Searches included the following keywords as search terms: *soccer* OR *football* AND *injury*, in combination with *fixture congestion, congestion, congested,* and *match congestion.* Additionally, reference lists of the articles retrieved were assessed for any additional relevant studies, and articles that were already known to the authors but not identified in the searches were further included. All articles were saved and duplicates removed using the reference manager software EndNote (EndNote X9; Thomson Reuters©, New York, NY, USA). Following the removal of duplicates, articles were screened firstly by title, followed by abstract, and finally the remaining full texts were examined for their suitability. If there were any discrepancies between the authors, then a third author (LDH) arbitrated the disagreement. All articles, and their reasons for omission, can be viewed on the Open Science Framework (https://osf.io/4f6t9).

### Assessment of Methodological Quality

The methodological quality of the studies included in this systematic review was evaluated using the quality assessment tool for observational cohort and cross-sectional studies (https://www.nhlbi.nih.gov/health-topics/study-quality-assessment-tools). The quality of each methodology was assessed by two authors (RMP and BL) using the 14-item assessment stated previously. Each item was assessed using three descriptions: yes (was included in the article), no (was not included in the article), or other (cannot determine, not applicable, not reported). As intended by the assessment tool, the items were not simply tallied to arrive at a summary of the quality of the studies. Instead, these items provided a focus on the key concepts for evaluating the internal validity of the study. Following the assessment, the quality of each study was determined as either *good, fair* or *poor* by each assessor*.*

### Data Extraction

Study characteristic information and data extraction were conducted by two authors (RMP and BL). If there were any discrepancies between the authors, then a third author (LDH) cross-checked the discrepancies. The study characteristics and associated data were grouped into three categories: (1) general study descriptors (e.g., authors, year of publication and study design); (2) description of the study population (e.g., sample size and level of play); and (3) epidemiological data (e.g., total injury incidence, injury incidence of specific anatomical locations and types of injury, the incidence of injury during specific match timings, and injury layoff durations). Data were extracted in relation to match injuries, training injuries, and overall injuries during the respective congested and non-congested periods. Based on the inclusion criteria of the current study, fixture congestion was defined as ≤ 4 days separating matches, with non-congested periods being games with > 4 days interspersing subsequent matches. Where possible, data are reported as mean ± standard deviation (SD) and 95% confidence intervals (CIs). *P*-values and effect sizes (Cohen’s *d*) were also extracted or calculated. Where specific *p* values were not included, these are reported as < 0.05 or > 0.05. Where SD or *d* values are not reported, CI values have been used to calculate SD using the formula stated in the Cochrane handbook [41], thus, in turn, allowing for *d* values to be calculated.

## Results

**Fig. 1:**
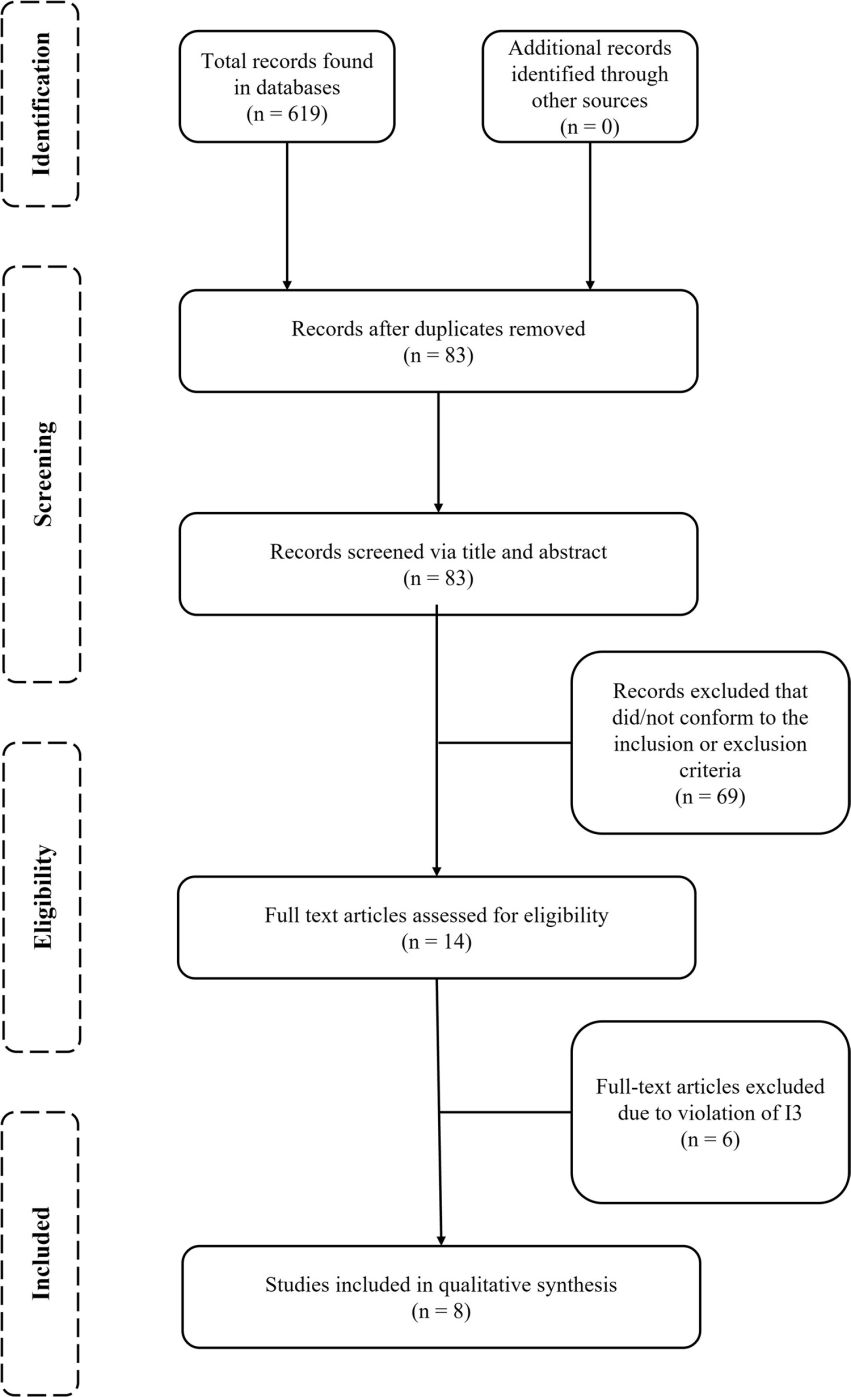
PRISMA flow diagram of the systematic procedure for article selection. Note: I3 = inclusion criteria 3, including relevant data regarding periods of fixture congestion on injuries during soccer match-play. Please refer to the Open Science Framework for all omitted articles, along with their reasons for omission (https://osf.io/4f6t9). *PRISMA* Preferred Reporting Items for Systematic Reviews and Meta-Analyses

A total of 619 records were identified following the electronic searches, with no additional articles located by the researchers during manual searches (Fig. 1). Following the omission of 536 duplicates, the remaining 83 titles and abstracts were screened. Sixty-nine articles were rejected as they did not meet the eligibility criteria, leaving 14 articles for full-text screening. Six articles were omitted following the full-text assessments, and eight studies were accepted and included in the systematic review.

### Study Characteristics

Characteristics of the included studies are presented in Table 1. Six studies adopted a prospective [1, 42–46], observational design, with two studies adopting a retrospective observational design [11, 47]. The included studies reported data on European [11, 47], French Ligue 1 [1, 42, 43, 46], Scottish Premiership [44] and Australian A-League teams [45]. Data collection periods ranged from a 26-day congested period [42], up to 14 seasons [47]. Of the included studies, the earliest articles were published in 2010 [44, 46], with the latest in 2020 [45]. Injury incidence and injury layoff durations were reported by eight and four studies, respectively, and will be considered in more detail in the subsequent sections. Other variables such as injury type, injury location, injury mechanism, and injury timing were reported sporadically (two or fewer studies) and inconsistently. Data related to these variables can be found within electronic supplementary Table S1 by interested readers.

**Table 1:** Summary of studies investigating injuries during periods of fixture congestion

References	Participants	Data collection methods	Injury definitions	Fixture congestion scenario	Measures specific to match-play injuries	Measures specific to training injuries	Measures specific to overall injuries
Carling et al. [46]	31 ± 2.5 professional players per season from a French Ligue 1 club	Data were collected prospectively across a 4-year period (2005–2006 season, up to and including the 2008–2009 season)	Considered time loss injuries, which resulted when a player was unable to take part in future soccer training or matches owing to physical complaints Injury classifications were made according to the Fuller et al. [48] injury classification consensus	76 congested matches 116 non-congested matches Congested periods were defined as < 3 days between matches Non-congested periods were defined as ≥ 4 days between matches	Injury rate (per 1000 h of match exposure) and injury severity	NA	NA
Dupont et al. [44]	32 professional players playing for the same top-level Scottish club	Data were collected prospectively across two seasons (2007–2008 and 2008–2009) Players were included if they completed ≥ 75 min in a match within the 4 days preceding a second match	Considered time loss injuries, which resulted when a player was unable to take part in future soccer training or matches owing to physical complaints Injury classifications were made according to the Fuller et al. [48] injury classification consensus	123 total matches that provided 116 congested match observations, and 130 non-congested match observations Congested matches were defined as ≤ 4 days between successive matches. Non-congested were played with a minimum of 6 days from the preceding match	Injury rates (per 1000 h of match exposure)	Injury rates (per 1000 h of training exposure)	Injury rates (per 1000 h of exposure) Although not statistically analysed, the study also presented the number of different mechanisms of injury (traumatic or overuse), number of locations of the injuries, number of types of injury, number of recurrent injuries (early, < 2 months; late, 2–12 months; or delayed, > 12 months), number of different severities of injury (slight, 1–3 days; minor, 4–7)
Carling et al. [42]	19 elite outfield players from the same French Ligue 1 club	Data were collected prospectively across a 26-day congested period during the 2009 season (8 matches in 26 days) 19 players participated in one or more matches within the congested period; 2 completed every game, 6 participated in every game either as a starter or substitute, and 8 participated in 75% or more of the total number of minutes played by the team. There was no exclusion criterion based on time played within matches across the 26 days	First team match injuries were considered, and inclusion criteria were those injuries leading to an outfield player being unable to fully participate in future training or matches (i.e., time-loss injury) The methods and definitions of injury used in the present study closely follow those recommended by the Fuller et al. [48] and Hägglund et al. [49] injury classification consensuses	Congested period comprised eight congested matches over a 26-day period In comparison, the study examined injuries occurring in the nine matches prior to the congested period and 13 matches in the post congested period	Injury rates (per 1000 h of exposure) and layoff duration (days) Although not statistically analysed, the data were reported for the number of different severities of injury, and the number of different injury locations	NA	NA
Bengtsson et al. [11]	27 professional teams competing in the highest level of European football between 2001 and 2012	Retrospective analyses of data collected during 11 consecutive seasons between 2001 and 2002 to 2011 and 2012 in a prospective cohort study known as the UEFA Elite Club Injury study (Ekstrand et al. [50])	Considered time loss injuries, which resulted when a player was unable to take part in future soccer training or matches owing to physical complaints Injury classifications according to the Fuller et al. [48] injury classification consensus	≤ 4 days between matches (4455 observations) were compared with > 6 days (2977 observations)	Overall injury rates (per 1000 h of match exposure) and injury rates for different injury types (muscular and ligamentous), and muscle injury locations (hamstring, quadriceps, adductor, calf)	NA	NA
Dellal et al. [43]	16 professional outfield players from the same French Ligue 1 club	Data were collected prospectively across 1 season (2011–2012) Participants were only included if they completed at least 75 min in all six congested matches within an 18-day period Only four players played in every game in all three congested periods	Considered time loss injuries, which resulted when a player was unable to take part in future soccer training or matches owing to physical complaints Injury classifications according to the Fuller et al. [48] injury classification consensus	18 congested match observations from the French league (*n* = 11) and cup (*n* = 1) and the UEFA champions league (*n* = 6) The number of non-congested games was not reported Data were recorded from three fixture congestion periods (six consecutive matches over an 18-day period), with each match separated by 3 days Congested periods were defined as ≥ 2 matches in a week. Non-congested periods were defined as one match per week	Injury rates (per 1000 h of match exposure) Although not statistically analysed, the injury circumstances were reported as the percentage suffered during training or match-play	Injury rates (per 1000 h of training exposure) Although not statistically analysed, the injury circumstances were reported as the percentage suffered during training or match-play	Injury rates (per 1000 h of exposure) Mean layoff duration (days) Injury severity (slight, 1–3 days; minor, 4–7 days; moderate, 8–28 days; major, more than 28 days) Although not statistically analysed, the mechanisms of injuries were reported as a percentage of injuries that were traumatic or overuse
Carling et al. [1]	14 professional players from the same French Ligue 1 club. These players provided 25 match observations over the collection period	Data were collected prospectively across a 6-year period (2009–2015) Injuries sustained both on national team duty and in club competitions were investigated	Time-loss injury resulting from playing football and leading to a player being unable to fully participate in future training or match-play independent of whether a training session took place on the day following injury or the player was selected to play in the next match Injury classifications according to the Fuller et al. [48] and Hägglund et al. [49] injury classification consensuses	Non-congested periods (total exposure time 724 h) vs: Congestion scenario 1: two successive matches separated by ≤ 3 days (total exposure time 269.2 h) Congestion scenario 2: three successive matches separated by ≤ 4 days (total exposure time 138.9 h)	Injury rates (per 1000 h of match exposure) were reported overall and for the injury causality, re-injuries, injury type, injury location, and within-match injury timing The injury layoff time (days) was also reported	NA	NA
Bengtsson et al. [47]	2672 players competing across 57 professional European teams from 16 countries all competing in the highest division in their respective countries	Retrospective analyses of data collected during 14 consecutive seasons between 2001 and 2002 to 2014 and 2015 in a prospective cohort study known as the UEFA Elite Club Injury study (Ekstrand et al. [50])	Considered time loss injuries, which resulted when a player was unable to take part in future soccer training or matches owing to physical complaints Injury classifications according to the Hägglund et al. [49] injury classification consensus	68,477 congested match observations Short-term fixture congestion defined as the total number of days elapsed since the player’s last recorded match exposure In relation to the definitions used in this review, data are reported as congested (< 3 and 4 days recovery between matches) and non-congested schedules (5, 6 days, and 7–10 days interspersing matches) The influence of previous match exposure duration was also considered (< 90 or ≥ 90 min of exposure in the previous match) The associations between long-term match congestion and injury rates were considered. Long-term match congestion was defined as the total hours of match exposure that players had been exposed to 30 days prior to an observation These observations were categorised in low (≤ 4.5 h), medium (> 4.5 to ≤ 7.5 h) or high (> 7.5 h) match congestion periods	Total and muscle injury rates (1000 h of match exposure)	NA	NA
Howle et al. [45]	28 professional players from the same Australia A-league	Data were collected prospectively over three seasons between 2012 and 2015 (total of 106 matches) Data were only included from players who completed > 75 min of match time in the single-match and within both matches of the multi-match weeks	Considered time loss injuries, which resulted when a player was unable to take part in future soccer training or matches owing to physical complaints Injury classifications according to the Fuller et al. [48] injury classification consensus	40 congested matches providing 86 multi-match week observations 37 single matches providing 214 observations Single-match weeks were defined as matches separated by > 6 days, and multi-match weeks were defined as matches separated by < 4 days	Match injury rates (1000 h of exposure) Although not statistically analysed, the total number of match injuries was also reported	Training injury rates (1000 h of exposure) Although not statistically analysed, the total number of training injuries was also reported	Overall injury rates (1000 h of exposure) Although not statistically analysed, the total number of overall injuries and number of contact and non-contact injuries were also reported

*NA* not available

### Match, Training, and Overall Injury Incidence

All eight studies reported data on total match injury incidence (Table 2). In all studies, the injury incidence during match play was higher during periods of fixture congestion; however, the difference was only significant in five studies [1, 11, 43–45]. Three studies [43–45] reported data on total injury incidence during training (Table 2), with two of these studies [44, 45] reporting significantly increased total training injury incidence during periods of fixture congestion. In contrast, Dellal et al. [43] reported significantly reduced total training injury incidence during congested periods. Three studies [43–45] reported data on overall (match and training) injury incidence (Table 2). Significantly increased overall injury incidence was reported during periods of fixture congestion in two studies [44, 45]; however, Dellal et al. [43] reported no significant difference in overall injury incidence between congested and non-congested periods.

**Table 2:** Summary of match, training and overall injury incidence during congested and non-congested periods from the included studies

References	Injury incidence during match-play (per 1000 h)				Injury incidence during training (per 1000 h)		Injury incidence overall (per 1000 h)	
	Congested		Non-congested		Congested	Non-congested	Congested	Non-congested
Carling et al. [46]	45.0 ± 54.6		37.7 ± 48.4 (*p* = 0.406; *d* = − 0.1)					
Dupont et al. [44]	**97.7 (CI 76.1–119.2)**		**19.3 (CI 11.9–26.7)** **(*p* < 0.05; *d* = − 1.24)**		**8.3 (CI 5.2–11.4)**	**2.5 (CI 1.6–3.4)** **(*p* < 0.05; *d* = − 0.65)**	**25.6 (CI 20.8–30.5)**	**4.2 (CI 3.0–5.1)** **(*p* < 0.01; *d* = − 1.56)**
Carling et al. [42]	50.3		49.8 (*p* = 0.94)					
Bengtsson et al. [11]								
League	**29.0**		**26.6 (RR 1.09, CI for RR 1.00–1.18)** **(*p* = 0.045)**					
UCL	33.0		27.1 (RR 1.22, CI for RR 0.85–1.75) (*p* = 0.290)					
EL	24.7		37.9 (RR 0.65, CI for RR 0.41–1.03) (*p* = 0.064)					
Other	27.8		23.6 (RR 1.18, CI for RR 0.94–1.47) (*p* = 0.153)					
Dellal et al. [43]	**43.3 (CI 33.3–57.5)**		**18.6 (CI 16.3–21.3)** **(*p* < 0.001; *d* = − 1.07)**		**4.6 (CI 3.2–5.8)**	**14.6 (CI 12.2–17.1)** **(*p* < 0.001; *d* = 1.92)**	14.4 (CI 13.7–14.9)	15.6 (CI 15.1–16.3) (*p* > 0.05; *d* = 0.75)
Carling et al. [1]								
2-match congestion	70.6 (CI 39.0–102.0**)** (*p* > 0.05; *d* = 0.39)		**47 (CI 31.0–63.0)**					
3-match congestion	**93.6 (CI 43.0–144.0)** **(*p* = 0.0345; *d* = 0.26)**							
Bengtsson et al. [47]	≤ 3 days	25.2 (CI 23.7–26.7)	5 days	24.3 (CI 23.7–26.7) (≤ 3 days: *p* > 0.05; *d* = − 0.02) (4 days: *p* > 0.05; *d* = 0.01)				
	4 days	25.0 (CI 23.5–26.5)	6 days	23.7 (CI 21.6–26.1) (≤ 3 days: *p* > 0.05; *d* = − 0.02) (4 days: *p* > 0.05; *d* = − 0.02)				
			≥ 7 days	23.9 (CI 22.6–25.3) (≤ 3 days: *p* > 0.05; *d* = − 0.03) (4 days: *p* > 0.05; *d* = − 0.02)				
Howle et al. [45]	**50.3 (CI 41.4–56.5)**		**44.8 (CI 33.1–56.5)** **(*p* < 0.05; *d* = 0.15)**		**16.9 (CI 11.7–22.1)**	**6.7 (CI 4.0–9.4)** **(*p* < 0.05; *d* = 0.68)**	**33.7 (CI 25.1–42.3)**	**15.6 (CI 10.8–20.4)** **(*p* < 0.05; *d* = 0.71)**

Data are expressed as mean ± standard deviation unless otherwise stated. Significant differences and associated Cohen’s *d* effect sizes (*d*) are shown in bold

*CI* 95% confidence interval, *RR* risk ratio, *UCL* UEFA Champions League, *EL* Europa League

### Injury Layoff Duration

Injury layoff times were reported within four studies [1, 42, 43, 46] (Table 3). Carling et al. [42] and Dellal et al. [43] both reported significant reductions in average layoff times during periods of fixture congestion compared with non-congested periods; however, Carling et al. [1, 46] reported no significant change in injury layoff time between congested and non-congested periods.

**Table 3:** Summary of layoff times during congested and non-congested periods from the included studies

References	Layoff duration (days)		
	Congested		Non-congested
Carling et al. [46]	15 ± 25		15 ± 28 (*p* = 0.730; *d* = 0.00)
Carling et al. [42]	**2.0 ± 1.5**		**7.9 ± 14.6 (*p* = 0.043; *d* = 0.57)**
Dellal et al. [43]	**9.5 ± 8.8**		**17.5 ± 29.6 (*p* = 0.012; *d* = 0.5)**
Carling et al. [1]	Scenario 1: 6.2 ± 3 (*p* = 0.523; *d* = − 0.17)	Scenario 2: 4.3 ± 3 (*p* = 0.145; *d* = − 0.62)	6.9 ± 2.9

Data are expressed as mean ± standard deviation unless otherwise stated

Significant differences and associated Cohen’s *d* effect sizes (*d*) are shown in bold

### Methodological Quality of Studies

The quality assessment results are presented in Table 4. Six studies were considered to be of ‘*good*’ quality [1, 42–45, 47], with the remaining two studies classified as having ‘*fair*’ quality [11, 46]. None of the studies satisfied all 14 criteria. Criteria 5 (‘was a sample size justification, power description, or variance and effect estimates provided?’) and 14 (‘were key potential confounding variables measured and adjusted statistically for their impact on the relationship between exposure[s] and outcome[s]?’) were not addressed in the included studies. Criteria 12 (‘were the outcome assessors blinded to the exposure status of participants?’) and 13 (‘was loss to follow-up after baseline 20% or less’) were not deemed applicable to any of the included studies. Three studies [11, 46, 47] failed to report Criterion 3 (‘was the participation rate of eligible persons at least 50%?’).

**Table 4:** Quality criteria for the quality assessment tool for observational cohort and cross-sectional studies

Criteria/included studies	Dupont et al. [44]	Carling et al. [46]	Carling et al. [42]	Dellal et al. [43]	Bengtsson et al. [11]	Carling et al. [1]	Bengtsson et al. [47]	Howle et al. [45]
1. Was the research question or objective in this paper clearly stated?	Y	Y	Y	Y	Y	Y	Y	Y
2. Was the study population clearly specified and defined?	Y	Y	Y	Y	Y	Y	Y	Y
3. Was the participation rate of eligible persons at least 50%?	Y	NR	Y	Y	NR	Y	NR	Y
4. Were all the subjects selected or recruited from the same or similar populations (including the same time period)? Were inclusion and exclusion criteria for being in the study prespecified and applied uniformly to all participants?	Y	Y	Y	Y	Y	Y	NR	Y
5. Was a sample size justification, power description, or variance and effect estimates provided?	Y	N	N	N	Y	N	N	N
6. For the analyses in this paper, were the exposure(s) of interest measured prior to the outcome(s) being measured?	Y	Y	Y	Y	Y	Y	Y	Y
7. Was the timeframe sufficient so that one could reasonably expect to see an association between exposure and outcome if it existed?	Y	Y	Y	Y	Y	Y	Y	Y
8. For exposures that can vary in amount or level, did the study examine different levels of the exposure as related to the outcome (e.g., categories of exposure, or exposure measured as a continuous variable)?	Y	Y	Y	Y	Y	Y	Y	Y
9. Were the exposure measures (independent variables) clearly defined, valid, reliable, and implemented consistently across all study participants?	Y	Y	Y	Y	Y	Y	Y	Y
10. Was the exposure(s) assessed more than once over time?	Y	Y	CD	Y	Y	Y	Y	Y
11. Were the outcome measures (dependent variables) clearly defined, valid, reliable, and implemented consistently across all study participants?	Y	N	Y	Y	Y	Y	Y	Y
12. Were the outcome assessors blinded to the exposure status of participants?	NA	NA	NA	NA	NA	NA	NA	NA
13. Was loss to follow-up after baseline 20% or less?	NA	NA	NA	NA	NA	NA	NA	NA
14. Were key potential confounding variables measured and adjusted statistically for their impact on the relationship between exposure(s) and outcome(s)?	N	N	N	N	Y	N	N	N
Quality rating	Good	Fair	Good	Good	Fair	Good	Good	Good

*CD* cannot determine, *N* no, *NA* not applicable, *NR* not reported, *Y* yes

## Discussion

The purpose of this study was to conduct a systematic review assessing the influence of a congested fixture schedule on injuries in professional male soccer. The findings suggest that periods of fixture congestion expose players to increased injury incidence, although layoff duration was typically lower during congested periods compared with non-congested periods. The current findings have implications for practitioners regarding the management, periodisation, monitoring, and design of training and competition schedules. Through the systematic organisation of the literature, researchers are guided where to direct future research efforts into injury epidemiology during fixture-congested periods.

### Total Match Injury Incidence

The studies included in this review mostly demonstrate that periods of fixture congestion expose players to an increased incidence of injury [1, 11, 43–45]. Although somewhat speculative, the studies [1, 42, 43, 46] conducted with teams competing in the French Ligue 1 offer a potential consideration of how injury incidence may be influenced by contemporary evolutions in match-play demands, and how these increased demands may result in increased injury incidence during more prolonged periods.

Previous research in the English Premier League [51] identified that the match demands increased in their sample between 2009 and 2014, suggesting contemporary match-play is characterised by more intense and demanding activity profiles. Although this study was specific to the increased intensity observed in the English Premier League, a similar increase in intensity could have feasibly occurred across other leagues. Interestingly, no differences in injury incidence were observed between congested and non-congested periods in the studies conducted between 2005 and 2010 on fixture congestion. However, more contemporary work (data collected between 2009 and 2012) did identify that congested match schedules increase injury incidence during both multi-match weekly microcycles and more prolonged periods of match congestion. This increased injury occurrence may be related to fatigue-induced reductions in a player’s capacity to cope with the maintenance of physical performance.

Although a recent meta-analysis indicates that players can maintain physical output during fixture-congested schedules [2], it is possible that a reduction in a player’s locomotor efficiency occurs [31]. Fatigue-induced modifications in running kinematics may be attributed to a change in musculotendon unit stiffness and a reduced lower extremity motor control [52], compromising passive and dynamic stability [53], possibly increasing injury propensity to ligaments and passive joint structures. An investigation found that the increment in sprint distance covered over a 1-min period increased the risk of muscle injury during match-play [32]. However, although fatigue may be increased during fixture-congested periods, and the risk of injury during high-volume sprinting might exacerbate injury risk, the aetiology of how a sprinting-related muscle injury might occur is unclear. Therefore, future research should assess the causal mechanisms associated with an increased injury risk during periods of short-term fixture congestion [10].

The observation of increased injury incidence during congested multi-match and single-match weekly microcycles has also been reported from elite Scottish and Australian soccer. The study in Scottish soccer [44] was conducted with data recorded between 2007 and 2009 and, as such, this falls within the period when the French Ligue 1 data do not support the notion of increased match injury incidence during congested schedules [1, 42, 43, 46]. The discrepancies in these findings may be due to varying styles of play and practices that are adopted across respective leagues and competitions.

To overcome these potential confounding factors when comparing data across leagues, research conducted by Bengtsson et al. [11, 47] collated injury data from clubs playing at the elite level across several leagues. Interestingly, regarding the data collated between 2001 and 2012 and considered across different competition types (league, domestic cups, UEFA Champions League and Europa League matches) significantly higher injury incidence was observed in congested league matches, but not UEFA Champions League and Europa League matches, when compared with non-congested matches within these competition formats. A follow-on study by the same group, but this time including data up to and including the 2015 season, identified no differences in total match injury incidence between congested and non-congested matches. These two studies, therefore, seem to suggest that the observed response may be specific to the type of competition and practices that are put in place regarding the demands of these different competitions. Additionally, more contemporary data across several elite leagues suggest that injury incidence is not significantly increased, thus offering a potential change in practices and knowledge of how to cope with the demands of congested match-play (recovery consideration, squad rotations, squad sizes, etc.). It could also be suggested that by pooling data across several leagues, the sensitivity of the data may have been reduced, thus limiting any potential responses that were a result of differences in styles of play. Again, these suggestions are somewhat speculative and would need to be considered further with additional research.

### Training and Overall Injury Incidence

Three studies considered the influence of congested periods on training and overall injury incidence [43–45]. Two of these studies identified increased training and overall injury incidence during congested periods [44, 45], whereas Dellal et al. [43] identified significantly lower training injury incidence and no difference in overall injury incidence between congested and non-congested periods. Dellal et al. [43] also reported that during congested schedules, the injury incidence observed during match-play was significantly higher than the incidence observed during training, with this response not observed during non-congested periods. These findings are therefore somewhat inconclusive, with these differences potentially due to methodological differences between studies. For example, the studies by Howle et al. [45] and Dupont et al. [44] ranged over multiple seasons, but for only two and three match-congested cycles. Where congested periods only comprise a small number of games, there may be less of a desire on behalf of the manager to alter training load demands. In comparison, the Dellal et al. [43] study comprised data from specific and prolonged periods of congestion (three occurrences of a six-match congested period). A plausible explanation for the findings from this study is that practitioners may reduce training loads and intensities and better consider recovery strategies. Likewise, players may subconsciously taper training efforts during congested schedules to maintain performance in the upcoming matches [15]. During congested schedules, tapering training intensities may lead to reduced injury incidence during training sessions [43]. However, this requires careful periodisation to optimise the balance between adaptation and recovery. For instance, if reductions in training intensities are adopted to aid recovery, practitioners need to ensure that training outside of these periods allows for players to develop the ability to cope with spikes in match load associated with congested fixture periods. Accordingly, practitioners need to consider whether they can progressively condition players to cope with spikes in match load during congested schedules and, if so, when this may be best developed and maintained.

### Injury Layoff Duration

Only four studies considered injury layoff durations [1, 42, 43, 46]. Of these studies, when up to three consecutive matches were considered in relation to congested schedules, no significant differences in layoff durations were observed between congested and non-congested schedules [1, 46]. However, when more prolonged periods of congestion were considered (six matches in 18 days and eight matches in 26 days), there were reduced layoff durations during congested periods when compared with non-congested periods [42, 43]. Therefore, these data appear to suggest that although injury incidence is higher during congested matches when compared with non-congested schedules, the layoff time for these injuries is reduced. Injuries suffered during congested schedules may result in reduced layoff durations and are not as burdensome as those outside of these periods, and although speculative, are potentially related to the gradual onset of ‘*niggles*’ because of a prolonged increase in match volumes and accumulated load [54]. Although speculative, it could also be suggested that during congested schedules, there might be an emphasis on more ‘important’ (knock-out) matches, in which coaches allow players to play who have not finished their rehabilitation. This in turn might be one factor leading to shorter layoff times during congested schedules compared with non-congested schedules.

It is also plausible that injuries may also be influenced by previous injuries [55], with the residual fatigue from the previous matches increasing the risk of recurrent injuries during short- and long-term match congestion. Therefore, players that have recently been absent due to injury must be closely monitored during congested schedules. Few studies have considered the trends in recurrent injury incidence during fixture congestion, and thus studies investigating whether previous injuries are risk factors for sustaining a secondary injury during congested scenarios appear warranted. With implications for increased specificity of (p)rehabilitation and recovery practices, additional research should focus on the injury types, locations, and associated mechanisms of injury. Although some studies reported specific data on injury types, locations, and associated mechanisms of injury, these data were only reported by two or fewer studies, and, as such, these data were not included in the manuscript. Additional detail on the reported injury types is included in electronic supplementary Table S1.

### Methodological Quality of the Studies

Of the eight included studies, six studies were considered to be of ‘*good*’ quality [1, 42–45, 47], with the remaining two studies classified as having ‘*fair*’ quality [11, 46]. The quality of each study was determined as either *good, fair,* or *poor* by each assessor. This was achieved by considering the internal validity of each study based on key concepts and not based on a tally of the 14-item assessment per se. A common theme across studies was the inconsistent approach and differing levels of detail pertaining to the methods. For example, some studies failed to consider between-match rotation strategies, match and training loads, and the differing recovery practices adopted across teams. Additionally, from a statistical perspective, some studies did not report specific *p* values or effect sizes for the differences in injury incidence between fixture congested and non-congested periods. Overall, these differences may explain the moderate heterogeneity observed between study metrics and make direct comparisons inherently difficult. Methodological consistency is important for future investigations into the impact of match congestion on time-loss injuries, notably in terms of the variables measured, reporting of data and the general approaches to study designs. Additional considerations are discussed in Sect. 4.7.

### Practical Applications

Fixture-congested periods appear to exacerbate injury propensity [1, 11, 43–45], but effective recovery intervention [7], substitution strategies and squad rotation [56] as well as tapering of training loads [57] may alleviate such concerns. When considering the use of replacements and team rotations, the results of most studies did not account for or report partial match player observations [11, 44–47, 58]. However, due to squad sizes, player availability, restrictions on substitutions, and tactical considerations, improved rotation and substitution approaches are not always feasible. The current findings of a potentially reduced layoff duration during congested schedules may also encourage coaches and managers to not rotate players as often as potential injuries may initially not be as burdensome. However, coaches should be aware that small and continuous tissue failure can be present prior to any pain or changes in function, and by not considering physical complaints that may initially seem somewhat innocuous, we may reduce our ability to prevent future overuse and more burdensome injuries in the future [54].

Furthermore, it must be noted that all the included studies were undertaken prior to the coronavirus disease 2019 (COVID-19) pandemic, with FIFA since authorising five substitutions per match, as opposed to three replacements pre-pandemic. Therefore, the influence of squad substitution strategies on injury incidence remains to be elucidated. Despite sports scientist and practitioner advice, coaches may not invest in squad rotation strategies during fixture congestion since inconsistent team selections may disrupt team dynamics and reduce tactical cohesion [2, 59]. Indeed, rotation approaches may result in reduced proficiency due to less skilled or ‘*match fit*’ players being involved and a subsequent potential increase in injury risk, especially if the rotated players are not appropriately conditioned to match demands. However, in accordance with the included studies, without using squad rotations, players who regularly play in congested schedules may have an elevated risk of injury. Nonetheless, preventive actions targeting training load, playing style, substitution strategies, and player monitoring might not be sufficient to protect players from injury. The FIFPRO 2022 Player and High-Performance Coach Surveys highlight the harm that fixture congestion is causing to players, both physically and mentally [3]. The increased number of matches players are competing in, across multiple competitions, and the associated travel involved, is pushing players beyond their limits, and potentially shortening their careers. The report, combined with the findings of this systematic review, highlight the need for a re-evaluation of existing competition scheduling and the introduction of changes by governing bodies to facilitate recovery and enhance player health and wellbeing. Until these changes are made, coaches and practitioners may need to rest players in matches where previously they would have played, to reduce risk of injury and ensure adequate recovery is achieved.

### Future Research Directions

The metrics discussed above in relation to injury incidence and layoff durations are those that have been consistently reported in the literature to date. Other variables (i.e., injury types, locations, layoff durations, and time occurrence) have been reported sporadically in a small number of studies; however, a greater understanding of these factors would provide a clearer indication as to how congestion influences injury risk and type. Nonetheless, research has demonstrated that large variations in injury types exist between continents [60]. Therefore, since most of the current epidemiological data were collected in UEFA clubs, researchers outside of Europe are encouraged to evaluate the global impact of fixture congestion. Laboratory-based work is also required exploring mechanisms of injury and potential intervention strategies to inform applied practices. In line with previous laboratory-based investigations [35, 37, 61], utilising soccer-specific exercise protocols may also enhance knowledge of the injury mechanisms associated with fixture-congested schedules. There is also a need to further consider the context of the congested schedules (competition type, inclusion of extra time, travel demands, etc.) and the potential additional demands these may place on the players.

Additional research is also required to assess if the current response is also observed in female and youth players alike. While congested schedules may not be as common in women’s soccer [2], female players may encounter fixture-congested periods in international tournaments. It could therefore be suggested that not only have females been shown to have high injury incidence [62–64] during traditional match scheduling, if they are also not as commonly exposed to congested games within their domestic seasons, then these congested tournaments may elicit even more of a risk of injury. A similar argument exists in youth soccer, which again is a population particularly susceptible to injury risk when compared with their senior counterparts [65], and, as such, we may also need to better consider how we safely develop their ability to cope with exposure to congested match play.

### Limitations

Several limitations were present in the current review. Although considered unlikely, it cannot be discounted that additional investigations complying with the selection criteria exist but were not identified. The selection of studies was limited to those published in English, with studies in other languages not considered. Although the methodological quality assessments were conducted by two independent reviewers, judging the quality of studies remains subjective and is often based on divergent interpretations; however, consensus was reached by all authors before inclusion. The small sample of studies eligible for inclusion in the systematic review may be reflective of the inconsistent methodological approach across studies. Research has demonstrated that using different injury surveillance approaches can influence injury data [66], with experts recommending that checklists are adhered to in order to enhance the consistency of reporting epidemiological data [67].

Furthermore, due to the large variance between measures and how they were reported between studies, a meta-analysis could not be carried out. For instance, there was a lack of homogeneity between the number of consecutive seasons and matches, teams (single or multiple) and player observations across studies, as well as the way in which certain metrics were reported and defined. Analyses were often performed for an entire team and did not account for the playing duration of individual players (i.e., some studies solely included players that competed for at least 75 min, but others did not specify) or the position they were playing. In fact, only one of the included studies compared injury incidence across different playing positions [46]. They identified significantly greater injury incidence (particularly muscle strains) for forwards compared with all other positions. Researchers and practitioners should aim to identify position-specific injury risk during fixture congestion, as this may assist with training prescription and coach decision making.

Additionally, inclusion of a temporal breakdown of within-match injury patterns rather than simply an overall number across matches could also provide more accurate insights. Modulating factors may have an influence on injury rates through increased physical output of players, including the quality of opposition and the score line (e.g., potentially increased distance covered and high-intensity running when playing a ‘higher level’ opponent, or when performing additional efforts when a team is behind in a match [68]). Therefore, accounting for these factors is required in future research.

## Conclusion

The present systematic review aimed to assess the influence of a congested fixture schedule on injuries in professional male soccer. The results suggest that overall injury risk is increased during matches completed within fixture-congested periods; however the layoff time is typically shorter. It was also identified that inconsistent responses were observed for training and overall injury incidence. Where differences exist in findings between studies, this may be attributable to differences in methods in relation to, but not limited to, the specific type of match (i.e., a league or cup match), differences in respective leagues and standards of competition, the congested match scheduling, and practices that are put in place by the respective clubs regarding the demands of these different matches and schedules. The findings provide actionable steps for practitioners regarding the planning and development of training and competition agendas, while providing a source of scientific evidence for governing bodies to elicit policy and cultural change to support athlete welfare and develop a more sustainable match calendar that promotes a player’s career longevity. Both epidemiological observations and mechanistic experiments are required to provide a holistic and comprehensive understanding of injury occurrence during fixture-congested schedules.

## Supplementary Information

Below is the link to the electronic supplementary material.

Supplementary file1 (DOCX 43 KB)

## Declarations

### Availability of data and material

The protocol was preregistered on the Open Science Framework prior to searches and analyses being completed (https://osf.io/8dsvw). The PRISMA 2020 checklist has been included via the following link (https://osf.io/p5awk). All articles, including reasons for omission following the removal of duplicates can be viewed on the Open Science Framework (https://osf.io/4f6t9).

## References

[1] Carling C, McCall A, Le Gall F, Dupont G (2016). The impact of short periods of match congestion on injury risk and patterns in an elite football club. Br J Sports Med.

[2] Julian R, Page RM, Harper LD (2021). The Effect of Fixture Congestion on Performance During Professional Male Soccer Match-Play: A Systematic Critical Review with Meta-Analysis. Sports Med.

[3] FIFPRO. Player and High Performance Coach Surveys. 2022 [cited 17 Aug 2022]. Available at: https://fifpro.org/media/u0wfy0ba/220610_fifpro_men_pwm_flash_en_digital-2.pdf.

[4] Carling C, McCall A, Le Gall F, Dupont G (2015). What is the extent of exposure to periods of match congestion in professional soccer players?. J Sports Sci.

[5] Gouttebarge V, Brink MS, Kerkhoffs GM (2019). The perceptions of elite professional footballers on the International Match Calendar: a cross-sectional study. Sci Med Footb.

[6] McCall A, Davison M, Andersen TE, Beasley I, Bizzini M, Dupont G (2015). Injury prevention strategies at the FIFA 2014 World Cup: perceptions and practices of the physicians from the 32 participating national teams. Br J Sports Med.

[7] Nédélec M, McCall A, Carling C, Legall F, Berthoin S, Dupont G (2012). Recovery in soccer. Sports Med.

[8] Ispirlidis I, Fatouros IG, Jamurtas AZ, Nikolaidis MG, Michailidis I, Douroudos I (2008). Time-course of changes in inflammatory and performance responses following a soccer game. Clin J Sport Med.

[9] Jones RN, Greig M, Mawéné Y, Barrow J, Page RM (2019). The influence of short-term fixture congestion on position specific match running performance and external loading patterns in English professional soccer. J Sports Sci.

[10] Carling C, Gregson W, McCall A, Moreira A, Wong DP, Bradley PS (2015). Match running performance during fixture congestion in elite soccer: research issues and future directions. Sports Med.

[11] Bengtsson H, Ekstrand J, Hägglund M (2013). Muscle injury rates in professional football increase with fixture congestion: an 11-year follow-up of the UEFA Champions League injury study. Br J Sports Med.

[12] López-Valenciano A, Ruiz-Pérez I, Garcia-Gómez A, Vera-Garcia FJ, Croix MDS, Myer GD (2020). Epidemiology of injuries in professional football: a systematic review and meta-analysis. Br J Sports Med.

[13] Windt J, Gabbett TJ (2017). How do training and competition workloads relate to injury? The workload—injury aetiology model. Br J Sports Med.

[14] Timmins RG, Bourne MN, Shield AJ, Williams MD, Lorenzen C, Opar DA (2016). Short biceps femoris fascicles and eccentric knee flexor weakness increase the risk of hamstring injury in elite football (soccer): a prospective cohort study. Br J Sports Med.

[15] McCall A, Carling C, Nedelec M, Davison M, Le Gall F, Berthoin S (2014). Risk factors, testing and preventative strategies for non-contact injuries in professional football: current perceptions and practices of 44 teams from various premier leagues. Br J Sports Med.

[16] Dunlop G, Ardern CL, Andersen TE, Lewin C, Dupont G, Ashworth B (2020). Return-to-play practices following hamstring injury: a Worldwide Survey of 131 Premier League Football Teams. Sports Med.

[17] Van Der Horst N, Backx F, Goedhart EA, Huisstede BM (2017). Return to play after hamstring injuries in football (soccer): a worldwide Delphi procedure regarding definition, medical criteria and decision-making. Br J Sports Med.

[18] Zambaldi M, Beasley I, Rushton A (2017). Return to play criteria after hamstring muscle injury in professional football: a Delphi consensus study. Br J Sports Med.

[19] Buckthorpe M, Wright S, Bruce-Low S, Nanni G, Sturdy T, Gross AS (2019). Recommendations for hamstring injury prevention in elite football: translating research into practice. Br J Sports Med.

[20] Hägglund M, Waldén M, Ekstrand J (2009). UEFA injury study—an injury audit of European Championships 2006 to 2008. Br J Sports Med.

[21] Ekstrand J, Hägglund M, Waldén M (2011). Injury incidence and injury patterns in professional football: the UEFA injury study. Br J Sports Med.

[22] Hägglund M, Waldén M, Ekstrand J (2009). Injuries among male and female elite football players. Scand J Med Sci Sports.

[23] Ekstrand J (2013). Keeping your top players on the pitch: the key to football medicine at a professional level. Br J Sports Med.

[24] Eirale C, Tol J, Farooq A, Smiley F, Chalabi H (2013). Low injury rate strongly correlates with team success in Qatari professional football. Br J Sports Med.

[25] Hägglund M, Waldén M, Magnusson H, Kristenson K, Bengtsson H, Ekstrand J (2013). Injuries affect team performance negatively in professional football: an 11-year follow-up of the UEFA Champions League injury study. Br J Sports Med.

[26] Henderson G, Barnes CA, Portas MD (2010). Factors associated with increased propensity for hamstring injury in English Premier League soccer players. J Sci Med Sport.

[27] Fousekis K, Tsepis E, Poulmedis P, Athanasopoulos S, Vagenas G (2011). Intrinsic risk factors of non-contact quadriceps and hamstring strains in soccer: a prospective study of 100 professional players. Br J Sports Med.

[28] van Dyk N, Bahr R, Whiteley R, Tol JL, Kumar BD, Hamilton B (2016). Hamstring and quadriceps isokinetic strength deficits are weak risk factors for hamstring strain injuries: a 4-year cohort study. Am J Sports Med.

[29] Ekstrand J, Hägglund M, Waldén M (2011). Epidemiology of muscle injuries in professional football (soccer). Am J Sports Med.

[30] Hawkins RD, Fuller CW (1999). A prospective epidemiological study of injuries in four English professional football clubs. Br J Sports Med.

[31] Barrett S, Midgley A, Reeves M, Joel T, Franklin E, Heyworth R (2016). The within-match patterns of locomotor efficiency during professional soccer match play: Implications for injury risk?. J Sci Med Sport.

[32] Gregson W, Di Salvo V, Varley MC, Modonutti M, Belli A, Chamari K (2020). Harmful association of sprinting with muscle injury occurrence in professional soccer match-play: a two-season, league wide exploratory investigation from the Qatar Stars League. J Sci Med Sport.

[33] Arnason A, Sigurdsson SB, Gudmundsson A, Holme I, Engebretsen L, Bahr R (2004). Risk factors for injuries in football. Am J Sports Med.

[34] Brink MS, Visscher C, Arends S, Zwerver J, Post WJ, Lemmink KA (2010). Monitoring stress and recovery: new insights for the prevention of injuries and illnesses in elite youth soccer players. Br J Sports Med.

[35] Tofari PJ, Kemp JG, Cormack SJ (2020). Measuring the response to simulated fixture congestion in soccer. Sci Med Footb.

[36] Silva J, Rumpf M, Hertzog M, Castagna C, Farooq A, Girard O (2018). Acute and residual soccer match-related fatigue: a systematic review and meta-analysis. Sports Med.

[37] Page RM, Marrin K, Brogden CM, Greig M (2019). Physical response to a simulated period of soccer-specific fixture congestion. J Strength Cond Res.

[38] Fowler P, Duffield R, Waterson A, Vaile J (2015). Effects of regular away travel on training loads, recovery, and injury rates in professional Australian soccer players. Int J Sports Physiol Perform.

[39] Abbott W, Brownlee TE, Harper LD, Naughton RJ, Clifford T (2018). The independent effects of match location, match result and the quality of opposition on subjective wellbeing in under 23 soccer players: a case study. Res Sports Med.

[40] Fullagar H, Skorski S, Duffield R, Meyer T (2016). The effect of an acute sleep hygiene strategy following a late-night soccer match on recovery of players. Chronobiol Int.

[41] Higgins JPT, Li T, Deeks JJ (editors). Chapter 6: choosing effect measures and computing estimates of effect. In: Higgins JPT, Thomas J, Chandler J, Cumpston M, Li T, Page MJ, Welch VA, editors. Cochrane handbook for systematic reviews of interventions version 6.3 (updated February 2022). Cochrane; 2022. Available from www.training.cochrane.org/handbook.

[42] Carling C, Le Gall F, Dupont G (2012). Are physical performance and injury risk in a professional soccer team in match-play affected over a prolonged period of fixture congestion?. Int J Sports Med.

[43] Dellal A, Lago-Peñas C, Rey E, Chamari K, Orhant E (2015). The effects of a congested fixture period on physical performance, technical activity and injury rate during matches in a professional soccer team. Br J Sports Med.

[44] Dupont G, Nedelec M, McCall A, McCormack D, Berthoin S, Wisløff U (2010). Effect of 2 soccer matches in a week on physical performance and injury rate. Am J Sports Med.

[45] Howle K, Waterson A, Duffield R (2020). Injury incidence and workloads during congested schedules in football. Int J Sports Med.

[46] Carling C, Orhant E, Le Gall F (2010). Match injuries in professional soccer: inter-seasonal variation and effects of competition type, match congestion and positional role. Int J Sports Med.

[47] Bengtsson H, Ekstrand J, Waldén M, Hägglund M (2018). Muscle injury rate in professional football is higher in matches played within 5 days since the previous match: a 14-year prospective study with more than 130 000 match observations. Br J Sports Med.

[48] Fuller CW, Ekstrand J, Junge A, Andersen TE, Bahr R, Dvorak J (2006). Consensus statement on injury definitions and data collection procedures in studies of football (soccer) injuries. Scand J Med Sci Sports.

[49] Hägglund M, Waldén M, Ekstrand J (2005). Injury incidence and distribution in elite football—a prospective study of the Danish and the Swedish top divisions. Scand J Med Sci Sports.

[50] Ekstrand J, Hägglund M, Kristenson K, Magnusson H, Waldén M (2013). Fewer ligament injuries but no preventive effect on muscle injuries and severe injuries: an 11-year follow-up of the UEFA Champions League injury study. Br J Sports Med.

[51] Barnes C, Archer D, Hogg B, Bush M, Bradley P (2014). The evolution of physical and technical performance parameters in the English Premier League. Int J Sports Med.

[52] Oliver JL, De Ste Croix MBA, Lloyd RS, Williams CA (2014). Altered neuromuscular control of leg stiffness following soccer-specific exercise. Eur J Appl Physiol.

[53] Hughes G, Watkins J (2006). A risk-factor model for anterior cruciate ligament injury. Sports Med.

[54] Whalan M, Lovell R, Sampson JA (2020). Do Niggles Matter?-Increased injury risk following physical complaints in football (soccer). Sci Med Footb.

[55] Hägglund M, Waldén M, Ekstrand J (2016). Injury recurrence is lower at the highest professional football level than at national and amateur levels: does sports medicine and sports physiotherapy deliver?. Br J Sports Med.

[56] Hills SP, Barwood MJ, Radcliffe JN, Cooke CB, Kilduff LP, Cook CJ (2018). Profiling the responses of soccer substitutes: a review of current literature. Sports Med.

[57] Reilly T, Drust B, Clarke N (2008). Muscle fatigue during football match-play. Sports Med.

[58] Carling C, McCall A, Le Gall F, Dupont G (2015). The impact of in-season national team soccer play on injury and player availability in a professional club. J Sports Sci.

[59] Folgado H, Duarte R, Marques P, Sampaio J (2015). The effects of congested fixtures period on tactical and physical performance in elite football. J Sports Sci.

[60] Tabben M, Eirale C, Singh G, Al-Kuwari A, Ekstrand J, Chalabi H (2022). Injury and illness epidemiology in professional Asian football: lower general incidence and burden but higher ACL and hamstring injury burden compared with Europe. Br J Sports Med.

[61] Page RM, Marrin K, Brogden CM, Greig M (2016). The biomechanical and physiological response to repeated soccer-specific simulations interspersed by 48 or 72 hours recovery. Phys Ther Sport.

[62] Clausen MB, Zebis MK, Møller M, Krustrup P, Hölmich P, Wedderkopp N (2014). High injury incidence in adolescent female soccer. Am J Sports Med.

[63] Del Coso J, Herrero H, Salinero JJ (2018). Injuries in Spanish female soccer players. J Sport Health Sci.

[64] Nilstad A, Andersen TE, Bahr R, Holme I, Steffen K (2014). Risk factors for lower extremity injuries in elite female soccer players. Am J Sports Med.

[65] Materne O, Chamari K, Farooq A, Tabben M, Weir A, Holmich P (2022). Shedding light on incidence and burden of physeal injuries in a youth elite football academy: a 4-season prospective study. Scand J Med Sci Sports.

[66] Tabben M, Whiteley R, Wik E, Bahr R, Chamari K (2020). Methods may matter in injury surveillance: “how” may be more important than “what, when or why”. Biol Sport.

[67] Bahr R, Clarsen B, Derman W, Dvorak J, International Olympic Committee Injury and Illness Epidemiology Consensus Group (2020). International Olympic Committee consensus statement: methods for recording and reporting of epidemiological data on injury and illness in sports 2020 (including the STROBE extension for sports injury and illness surveillance (STROBE-SIIS)). Orthop J Sports Med.

[68] Castellano J, Blanco-Villaseñor A, Alvarez D (2011). Contextual variables and time-motion analysis in soccer. Int J Sports Med.

