# Supplementary material for: The Effects of Fixture Congestion on Injury in Professional Male Soccer: A Systematic Review
Source: Sports Med. 2022 Dec 17;53(3):667–85. doi: 10.1007/s40279-022-01799-5 (PMC9758680; doi:10.1007/s40279-022-01799-5)
Supplement: Supplementary file 1 — Supplementary file1 (DOCX 43 KB) [file 40279_2022_1799_MOESM1_ESM.docx]

| **Table S1.** Additional findings that were not reported in the main manuscript due to ≤2 studies reporting the same data ↑, ↓, ↔ denote significant increase in, significant decrease in, and no significant difference, respectively. CI= 95% confidence intervals. UCL= UEFA Champions League. EL= Europa League | | | | |
| --- | --- | --- | --- | --- |
| **Reference** | **Match-play injury data** | **Training injury data** | **Overall Injury data** | **Additional findings** |
| Carling et al. [46] |  |  |  |  |
| Dupont et al. [44] |  |  |  |  |
| Carling et al. [42] |  |  |  |  |
| Bengtsson et al. [11] | Data is presented as congested vs non-congested injury incidence per 1000h. CI data is not included to aid clarity  Muscle injuries  League: 11.4 vs 10.3  UCL: 13.5 vs 11.4  EL: 8.9 vs 9.0  Other: 11.2vs 9.1  Ligament injuries  League: 5.2 vs 5.3  UCL: 5.7 vs 5.7  EL: 3.6 vs 4.0  Other: 5.1 vs 4.5  Specific muscular injuries in league matches  Hamstring: 5.74 vs 4.47  Quadriceps: 1.53 vs 0.85  Adductor: 8.9 vs 9.0  Calf: 11.2 vs 9.1 |  |  | ↑ muscle injury incidence in league matches completed with ≤ 4 days between matches vs. > 6 days.  ↑ ligamentous injuries in other cup games (mainly domestic cup matches) ≤ 4 days between matches vs. > 6 days  ↔ in muscle and ligamentous injuries during UCL and EL games ≤ 4 days between matches vs. > 6 days.  ↑ hamstring and quadricep injuries in league matches completed ≤ 4 days between matches vs. > 6 days |
| Dellal et al. [43] |  |  |  |  |
| Carling et al. [1] | Data is presented as scenario 1 vs. scenario 2 vs. non-congested and represents the injury incidence per 1000h. CI data not included to aid clarity  Reinjury rates:  3.7 vs 0 vs. 5.5  Injury type and location incidence:  Strains overall: 33.3 vs. 36 vs. 16.6  Hamstring: 22.3 vs. 28.8 vs. 11.1  Quadriceps: 0 vs. 7.2 vs. 1.4  Groin: 3.7 vs. 0 vs. 1.4  Calf: 0 vs. 0 vs. 4.1  Other: 7.4 vs. 0 vs. 0  Sprains overall:14.9 vs. 28.8 vs. 4.1  Ankle: 14.9 vs. 28.8 vs. 2.8  Knee: 0 vs. 0 vs. 1.4  Contusion: 14.9 vs. 12.8 vs. 19.4  Injury incidence associated with the cause of non-contact injuries:  Overall: 44.6 vs. 50.4 vs. 24.9  Acceleration: 7.4 vs. 5.5 vs. 0.0  COD: 14.9 vs. 21.6 vs. 2.8  Fall: 4 vs. 0 vs. 2.8  Kicking ball: 3.7 vs. 0 vs. 1.4  Landing: 0 vs. 0 vs. 1.4  Tackle: 3.7 vs 7.2 vs. 0  Unknown: 11.1 vs. 21.6 vs. 11.1  Injury incidence across time periods:  1^st^ half overall: 26.0 vs. 50.4 vs. 19.4  0-15 min: 4 vs. 14.4 vs 1.4  16-30 min: 11.1 vs. 21.6 vs. 9.7  31 min- Half time: 11.1 vs. 14.6 vs. 8.3  2^nd^ half overall: 37.1 vs. 28.8 vs. 19.4  45-60 min: 3.7 vs. 7.3 vs. 8.3  61-75 min: 7.4 vs. 7.3 vs. 2.8  76 min- end: 26.0 vs. 14.4 vs. 8.3  Unknown: 7.4 vs. 14.4 vs. 8.3 |  |  | ↑ injury incidence of sprains and specifically ankle sprains in scenario 2 vs. non-congested periods.  ↑ non-contact injury incidence because of change in direction actions in the ﬁnal match in scenario 2 vs. non-congested periods.  ↑ injury incidence in the ﬁrst-half of matches in Scenario 2 vs. the first half of match play in non-congested periods.  ↑ injury incidence in the last 15 minutes of matches in scenario 1 vs. non-congested match-play. |
| Bengtsson et al. [47] | CI data is not included to aid clarity. The data represents the injury incidence per 1000h  ≥ 90 min exposure in previous match (data presented as total injury incidence/ muscle injury incidence):  Congested scheduling:  ≤ 3 days: 24.7/9.7  4 days: 24.0/10.6  Non-congested scheduling:  5 days: 25.3/11.4  6 days: 23.0/7.6  7-10 days: 22.7/8.0  ≤ 90 min exposure in previous match (data presented as total injury incidence/ muscle injury incidence):  Congested scheduling:  ≤ 3 days: 26.1/10.6  4 days: 27.2/10.5  Non-congested scheduling:  5 days: 22.0/6.7  6 days: 25.6/8.6  7-10 days: 26.6/8.5  Muscle injury incidence in different muscle groups following a full match-exposure categorized dependent on short-term match congestion. The data is presented as injury incidence data for the Hip or groin/thigh/lower limb muscle respectively:  Congested scheduling:  ≤ 3 days: 2.4/5.9/1.1  4 days: 2.7/6.1/1.5  Non-congested scheduling:  5 days: 3.9/ 5.9/ 1.2  6 days: 1.9/4.6/0.7  7-10 days: 2.0/4.8/0.9  Low, medium, and high long-term match congestion. The data is presented as low/medium/high muscle injury incidence data respectively:  Congested scheduling:  ≤ 3 days: 10.6/10.3/9.0  4 days: 13/10.2/10.3  Non-congested scheduling:  5 days: 15.5/ 12.1/ 8.9  6 days: 8.7/7.7/6.2  7-10 days: 8.2/8.0/7.5 |  |  | ↓ muscular injury incidence with 6 (21%) and 7-10 days recovery (19%) between matches vs. ≤ 3 days.  ↔ in muscular injury incidence between 4- or 5-days rest vs. ≤ 3 days.  ↑ muscular injuries (18-22%) if previous exposure was ≥ 90 mins when ≤ 3 days vs. 6 and 7-10 days.  Players with < 90 mins exposure in previous match had ↓ muscle injuries (37%) when 5 days interspersed matches vs. 3 days.  ↑ hip/groin muscle injuries were observed when  matches were separated by 5 days compared with ≤3 when previous exposure was ≥ 90 mins  ↓ muscle injury incidence for medium long-term match congestion with 7-10 days recovery when compared with ≤ 3 days. No other differences were observed for total or muscular injury incidence when comparing across long term exposures. |
| Howle et al., [45] |  |  |  |  |
